# Anti-Enterovirus 71 Agents of Natural Products

**DOI:** 10.3390/molecules200916320

**Published:** 2015-09-09

**Authors:** Liyan Wang, Junfeng Wang, Lishu Wang, Shurong Ma, Yonghong Liu

**Affiliations:** 1 College of Chinese Medicinal Materials, Jilin Agricultural University, Changchun 130118, China, E-Mail: wly8992@126.com; 2 Key Laboratory of Tropical Marine Bio-Resources and Ecology, Guangdong Key Laboratory of Marine Materia Medica, RNAM Center for Marine Microbiology, South China Sea Institute of Oceanology, Chinese Academy of Sciences, Guangzhou 510301, China; E-Mail: wangjunfeng@scsio.ac.cn; 3 Jilin Provincial Academy of Chinese Medicine Sciences, Changchun 130021, China; E-Mail: wls6856@163.com; 4 Endoscopy Center, China-Japan Union Hospital, Jilin University, Changchun 130021, China; 5 South China Sea Bio-Resource Exploitation and Utilization Collaborative Innovation Center, Guangzhou 510301, China

**Keywords:** anti-enterovirus 71, natural products, antiviral activity

## Abstract

This review, with 42 references, presents the fascinating area of anti-enterovirus 71 natural products over the last three decades for the first time. It covers literature published from 2005–2015 and refers to compounds isolated from biogenic sources. In total, 58 naturally-occurring anti-EV71 compounds are recorded.

## 1. Introduction 

Human enterovirus 71 (EV71) is one of the major causative agent of hand, foot and mouth disease (HFMD) in infants and children aged <10 years, and is a positive-sense, single-stranded RNA virus in the genus Enterovirus (family Picornavirus). About 7400-base genomes contain a single long open reading frame (ORF) with untranslated regions (UTR) at the 50 and 30 ends and a variable length poly-A tail at the terminus of the 30UTR. The ORF is divided into three consecutive parts, P1, P2 and P3. The viral RNA encodes a large polyprotein which is cleaved by virus-encoded and host proteases to produce the mature proteins. EV71 is divided into four genotypes, A, B, C and D. The most common form of EV71 infection is HFMD. Initial signs and symptoms include fever, headache, sore throat and a flu-like syndrome. Within a few days, patients develop painful ulcerated lesions in the nose, mouth and throat, accompanied by a rash that typically affects the hands and feet. In addition to HFMD, EV71 infection may involve the upper respiratory tract, the gastrointestinal tract, the cardiovascular system and the central nervous system. Neurological diseases range in severity from aseptic meningitis to acute flaccid paralysis and fatal encephalitis [1].

EV71 has emerged as a clinically important neurotropic virus that can cause acute flaccid paralysis and encephalitis, leading to cardiopulmonary failure and death. This illness has caused mortalities in large-scale outbreaks in the Asia-Pacific region in recent years, with widespread occurrence in China [2,3], creating a need to develop new anti-EV71 agents. Accordingly, exploring anti-EV71 agents is important. Several strategies have been used to develop antiviral drugs on the basis of the molecular characteristics of the virus [4]. Currently, no direct targeting vaccines or antivirals are available to treat severe EV71 infections.

At present, the prevention of EV71 epidemics mainly depends on public surveillance, and more effort should be made to develop drugs to conquer EV71 infections. Many compounds from various pharmacological medicinal plants have been extensively researched, not only for their potential inhibitory properties against virus invasion, but also for their low toxicity in cells. Therefore, it is essential to identify novel anti-EV71 agents as candidates for further research and optimization.

This review covers the literature from 2005–2015 and describes 58 compounds from 42 articles. Several reviews have dealt with anti-enterovirus 71 synthetic small molecules [1,2,3,4,5,6,7]. This review gives an overview of 58 natural products (NPs), semi-synthetic NPs and NP-derived compounds from natural sources during the last decade (2005–2015) which exhibit anti-enterovirus 71 (EV71) activities. To date, no review has focused primarily on anti-EV71 natural products.

## 2. Flavonoids

Flavonoids are widely distributed natural products with broad biological and pharmacological activities, including anti-EV71 activity.

### 2.1. Flavonols

**Figure 1:**
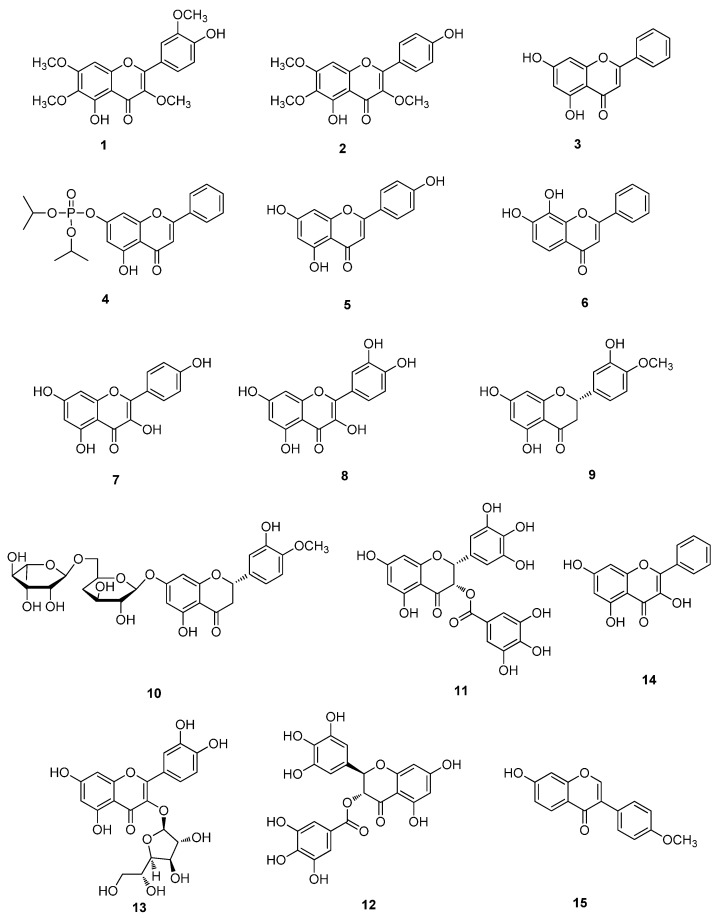
Flavonoids of **1**–**15**.

Chrysosplenetin (**1**) and pendulentin (**2**), two flavonols (Figure 1) isolated from the leaves of *Laggera pterodonta*, showed strong activity against EV71 in Vero and RD cell-based infection systems and inhibited viral RNA replication [8]. Vero cells are isolated from African green monkey and used as host cells for growing viruses.

### 2.2. Flavones

The anti-EV71 effect of chrysin (5,7-dihydroxyflavone, **3**), a natural flavonoid found in many plants, chrysin (**3**) could suppress viral 3Cpro activity. Replication of viral RNA and production of viral capsid protein and the infectious virion were strongly inhibited by chrysin (**3**), without noticeable cytotoxicity. Diisopropyl chrysin-7-yl phosphate (CPI, **4**), the phosphate ester for chrysin, was generated through a simplified Atheron-Todd reaction to achieve stronger anti-viral activity. CPI (**4**) was also able to bind with and inhibit viral 3Cpro activity *in vitro*. CPI (**4**) demonstrated more potent antiviral activity against EV71 [9]. Apigenin (**5**) blocks EV71 infection by disrupting viral RNA. The EC_50_ value was determined to be 10.3 µM, while the CC_50_ was 79.0 µM. Accordingly, suppression of hnRNP A1 and A2 expression markedly reduced EV71 infection [10]. 7,8-dihydroxyflavone (**6**), kaempferol (**7**), quercetin (**8**), hesperetin (**9**) and hesperidin (**10**) exhibited more than 80% of cell survival and inhibition of EV71 infection; among them, only 7,8-dihydroxyflavone (**6**), kaempferol (**7**) and hesperetin (**9**) showed 40% of viral IRES activity, kaempferol (**7**) interfered with EV71 virus replication, may change the composition of IRES associated trans-acting factors, and affect IRES function [11]. Tea polyphenols epigallocatechin gallate (EGCG, **11**) and gallocatechin gallate (GCG, **12**) potently inhibited replication of EV71. The antiviral effect correlated with the antioxidant activity of polyphenol. EV71 infection increased oxidative stress. With treatment of EGCG (**11**), reactive oxygen species (ROS) generation was significantly reduced. EV71 replication was enhanced in glucose-6-phosphate dehydrogenase deficient cells, and such enhancement was significantly reversed by EGCG (**11**) [12].

### 2.3. Flavone Glycosides

Luteolin (**13**), galangin (**14**) and quercetin (**8**) were identified as potential inhibitors of EV71 infection by reporter virus-based assays and cell viability-based assays, among which luteolin (**13**) exhibited the most potent inhibition of viral infection. The EC_50_ value of luteolin (**13**) was about 10 µM. Luteolin (**13**) targeted the post-attachment stage of EV71 infection by inhibiting viral RNA replication [13].

### 2.4. Isoflavones

**Figure 2:**
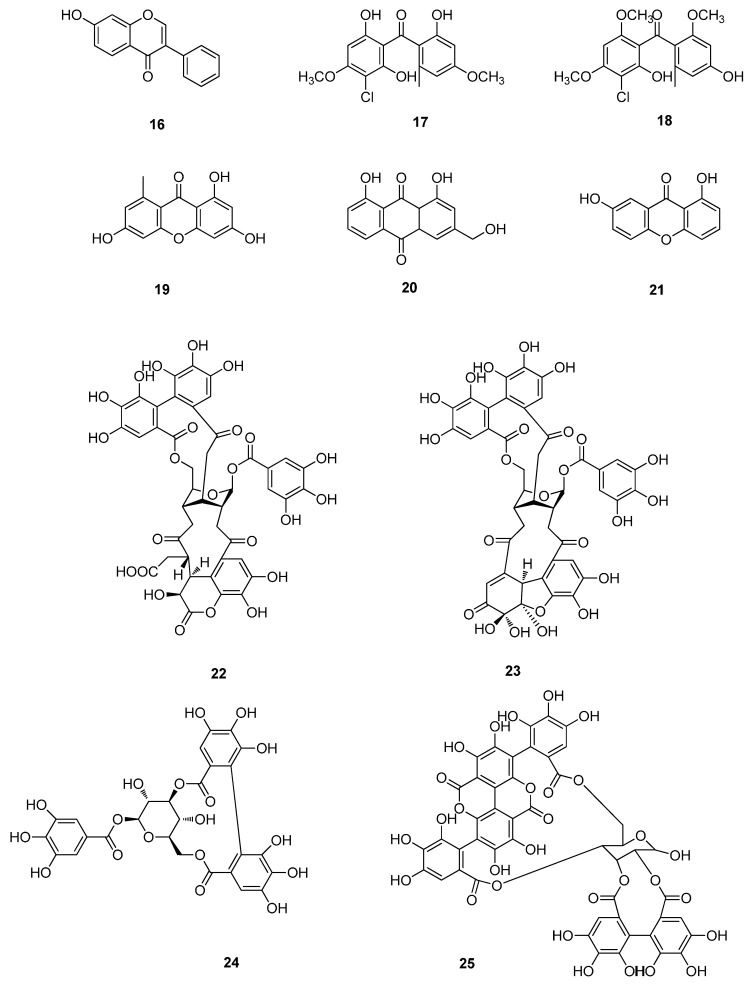
Xanthones and polyphenols of **16**–**25**.

The activation of ERK, p38 and JNK signal cascade in host cells has been demonstrated to up-regulate EV71-induced cyclooxygenase-2 (COX-2)/prostaglandins E-2 (PGE2) expression which is essential for viral replication. Formononetin (**15**) could reduce EV71 RNA and protein synthesis, inhibit COX-2 expression and PGE2 production via MAPKs pathway, and suppress the activation of ERK, p38 and JNK signal pathways [14]. 7-Hydroxyisoflavone (**16**) exhibited strong antiviral activity against three different EV71 strains. 7-Hydroxyisoflavone (**16**) could reduce EV71 viral RNA and protein synthesis in a dose-dependent manner (Figure 2). 7-Hydroxyisoflavone (**16**) showed significant antiviral activity in infected Vero cells. 7-hydroxyisoflavone (**16**) acted at an early stage of EV71 replication [15].

### 2.5. Xanthones

Three xanthone derivatives, stachybogrisephenone B (**17**), grisephenone A (**18**) and 3,6,8-trihydroxy-1-methylxanthone (**19**), were isolated from the cultures of sponge-derived fungus *Stachybotry* sp. HH1 ZDDS1F1-2 showing antiviral activities against EV71 [16]. The antiviral activity of aloe-emodin (**20**) against EV71 was evaluated using dose- and time-dependent plaque reduction assays in HL-CZ cells and TE-671 cells. The IC_50_ of aloe-emodin ranged from 0.14–0.52 µg/mL for EV71. Aloe-emodin (**20**) showed potent virus inhibitory activities against HL-CZ cells, inhibited EV71 replication via IFN signaling responses [17]. An acetone extract of the leaves of *Garcinia oblongifolia* showed antiviral activity against EV71 using a CPE inhibition assay. Bioassay-guided fractionation yielded auxanthone (**21**) exhibited significant anti-EV71 activity *in vitro*, with an IC_50_ value of 12.2 µM. In addition, the selectivity indices of **21** was 3.0 in Vero cells [18].

## 3. Polyphenols

### 3.1. Tannins 

A hydrolyzable tannin, chebulagic acid (**22**), was isolated from *Terminalia chebula* fruits found in Asia and Africa. Treatment with chebulagic acid (**22**) reduced the viral cytopathic effect on rhabdomyosarcoma cells with an IC_50_ of 12.5 μg/mL. Chebulagic acid (**22**) efficiently reduced mortality and relieve clinical symptoms through the inhibition of viral replication.[19] Geraniin (**23**) effectively inhibited virus replication in rhabdomyosarcoma cells with an IC_50_ of 10 µg/mL. Moreover, geraniin (**23**) treatment of mice that were challenged with a lethal dose of EV71 resulted in a reduction of mortality, relieved clinical symptoms, and inhibited virus replication in muscle tissues [20]. The corilagin (**24**) was a major component of *Phyllanthus urinaria* extract. Corilagin (**24**) inhibited EV71 infection *in vitro*. Corilagin (**24**) reduces the cytotoxicity induced by EV71 on Vero cells with an IC_50_ value of 5.6 µg/mL [21]. Punicalagin (**25**) reduced the viral cytopathic effect on rhabdomyosarcoma cells with an IC_50_ of 15 µg/mL, as well as reducing mortality and relieving clinical symptoms by inhibiting viral replication [22]. 

### 3.2. Polyphenols

**Figure 3:**
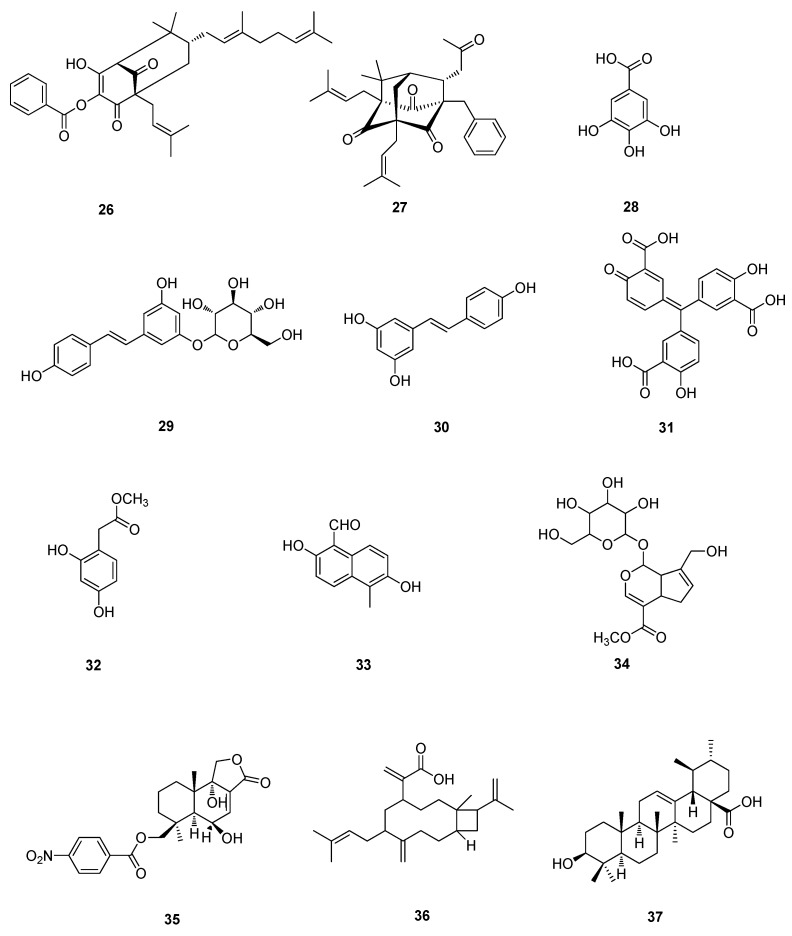
Polyphenols and terpenoids of **26**–**37**.

Two prenylated benzoylphloroglucinols, oblongifolins J (**26**) and M (**27**), were isolated from acetone extract of the leaves of *Garcinia oblongifolia*, exhibited significant anti-EV71 activity in *vitro*, with IC_50_ values of 31.1 and 16.1 µM, respectively (Figure 3). In addition, the selectivity indices of oblongifolins J (**26**) and M (**27**) were 1.5 and 2.4 in African green monkey kidney (Vero) cells, respectively [18]. Gallic acid (GA, **28**) isolated from *Woodfordia fruticosa* Kurz (family; Lythaceae) flowers exhibited a higher anti-EV71 activity than the extract of *W. fruticosa* flowers, with an IC_50_ of 0.76 µg/mL and no cytotoxicity at a concentration of 100 µg/mL [23]. Polydatin (**29**) and resveratrol (**30**) were major active components in *Polygonum cuspidatum*, and have antioxidant, anti-inflammatory and antitumor functions. Resveratrol (**30**) revealed strong antiviral activity on EV71, while polydatin (**29**) had weak effect. Resveratrol (**30**) could effectively inhibit the synthesis of EV71/VP1 and the phosphorylation of IKKα, IKKβ, IKKγ, IKBα, NF-κB p50 and NF-κB p65, increased secretion of IL-6 and TNF-α in EV71-infected rhabdosarcoma (RD) cells. Resveratrol (**30**) inhibited EV71 replication and cytokine secretion in EV71-infected RD cells [24]. Resveratrol (**30**) was nonpoisonous to Vero cells with an median toxic concentration (TC_50_) of 307.6 µM. Resveratrol (**30**) has an obvious inhibitory effect against EV 71 only before the cell infection by the virus (IC_50_ = 20.2 µM) [25]. Aurintricarboxylic acid (ATA, **31**) was found to be a potent inhibitor of EV71 replication with an EC50 of 2.9 μM. ATA (**31**) is able to effectively inhibit EV71 replication by interfering with the viral 3D polymerase [26]. Methyl 3,4-dihydroxyphenylacetate (**32**) has the inhibitory activity on the EV71 infection. The EV71 VP1 capsid protein expression levels were analyzed with Western blotting. **32** is able to inhibit EV71 replication in rhabdomyosarcoma (RD) cells. After incubating with the compound at a concentration of 0.01 µg/mL for 48 h, the level of EV71 vpl mRNA in RD cells decreased by (76.83 ± 2.47)%. **32** had low toxicity with a CC_50_ of 0.0726 µg/mL [27]. A naphthalene derivative, vaccinal A (**33**), was isolated from *Pestalotiopsis vaccinii* endogenous to the mangrove plant *Kandelia candel* (L.) Druce. It exhibited *in vitro* anti-EV71 with IC_50_ value of 19.2 µM [28].

## 4. Terpenoids

### 4.1. Monoterpenoid Glycoside

*Fructus gardeniae* greatly reduces anti-EV71 activity, resulting in significant decreases in EV71 virus yields, EV71 infections, and internal ribosome entry site activity. Geniposide (**34**), a primary *Fructus gardeniae* component, inhibited both EV71 replication and viral IRES activity. **34** blocks viral protein translation [29].

### 4.2. Sesquiterpenoids

A nitrobenzoyl sesquiterpenoid, 6β,9α-dihydroxy-14-*p*-nitrobenzoylcinnamolide (**35**) was isolated from extracts of marine-derived fungus *Aspergillus ochraceus* Jcma1F17, **35** showed antiviral activities against EV71 at 9.4 μM [30].

### 4.3. Sesterterpenoids

Raoulic acid (**36**), purified from a whole-plant extract of a New Zealand plant, *Raoulia australis*, was tested for antiviral activity in Vero cells and inhibited EV71 with an EC_50_ of less than 0.1 µg/mL and a CC_50_ of more than 65 µg/mL, giving it a therapeutic index >650 [31].

### 4.4. Triterpenoids

A large number of triterpenoids have been shown to have multiple biological activities.

#### 4.4.1. Triterpenoids

**Figure 4:**
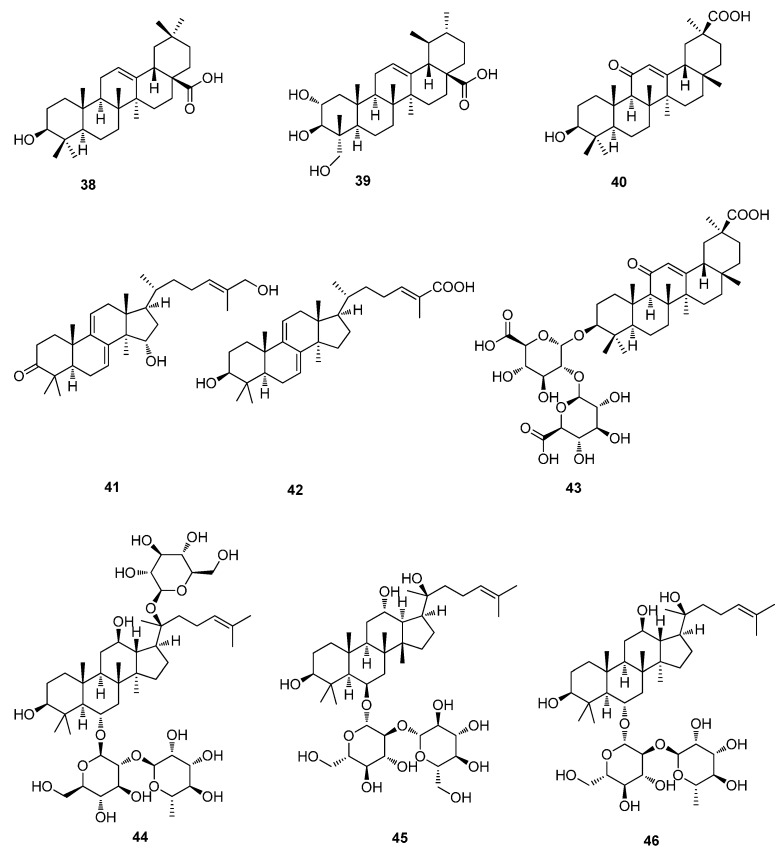
Triterpenoids of **38**–**46**.

Ursolic acid (**37**) is a triterpenoid purified from the aqueous extract of *Ocimum basilicum*, a herb commonly used in traditional Chinese medicine. Studies revealed post-infection inhibition of EV71 by lower doses of **37** [32]. Pentacyclic triterpenoids oleanolic acid (OA, **38**), asiatic acid (AA, **39**), ursolic acid (UA, **37**) and synthetic derivatives of 18-β-glycyrrhetinic acid (GA, **40**), exhibited inhibitory effects against EV71 (Figure 4) [33]. The antiviral activities of two *Ganoderma lucidum* triterpenoids, lanosta-7,9(11),24-trien-3-one,15,26-dihydroxy (GLTA, **41**) and ganoderic acid Y (GLTB, **42**), were demonstrated against EV71 infection. They display significant anti-EV71 activities without cytotoxicity in human rhabdomyosarcoma (RD) cells as evaluated by MTT cell proliferation assay. GLTA (**41**) and GLTB (**42**) prevent EV71 infection through interacting with the viral particle to block the adsorption of virus to the cells. GLTA (**41**) and GLTB (**42**) may bind to the viral capsid protein at a hydrophobic pocket (F site), and thus may block uncoating of EV71. GLTA (**41**) and GLTB (**42**) significantly inhibit the replication of the viral RNA (vRNA) of EV71 replication by blocking EV71 uncoating [34].

#### 4.4.2. Triterpenoids Glycosides

**Figure 5:**
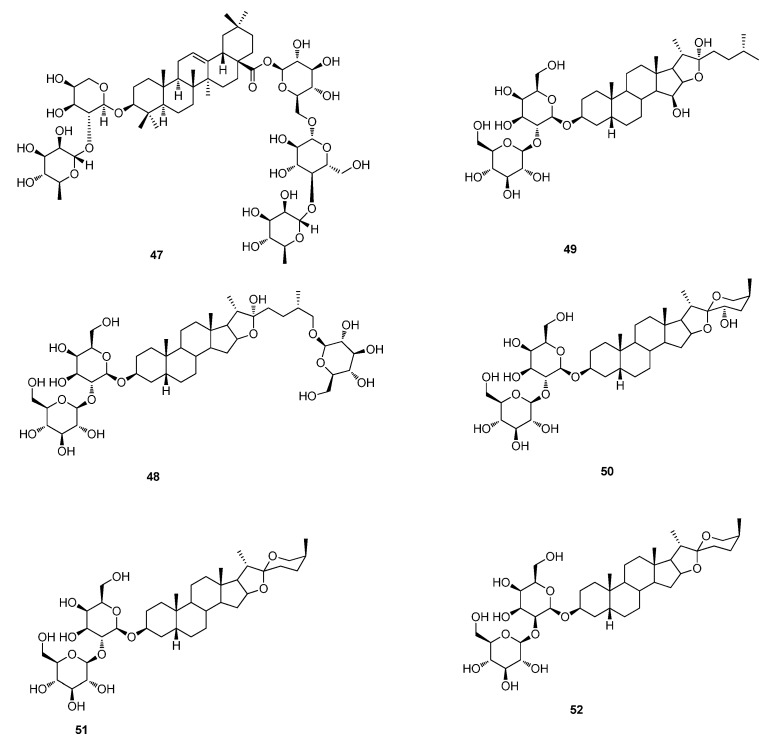
Triterpenoids and steroids of **47**–**52**.

Glycyrrhizic acid (**43**) is considered the principal component in *Glycyrrhiza* spp. with a wide spectrum of antiviral activity. **43** dose-dependently blocked viral replication of EV71. At 3 mM, **43** reduced infectious EV71 production by 2.2 logs. At 5 mM, EV71 production was reduced by 6.0 logs 4.0 logs [35]. Ginsenosides are the major components responsible for the biochemical and pharmacological actions of ginseng, and have been shown to have various biological activities. The antiviral activities of three protopanaxatriol (PT) type ginsenosides, R_e_ (**44**), R_f_ (**45**) and R_g2_ (**46**), were demonstrated against EV71. The antiviral efficacies of PT-type ginsenosides were comparable to those of ribavirin, a commonly used antiviral drug [36]. The antiviral activity of hederasaponin B (**47**) from *Hedera helix* against EV71 subgenotypes C3 and C4a was evaluated in vero cells. Hederasaponin B (**47**) showed potent antiviral activity against EV71 subgenotypes C3 and C4a (Figure 5). Hederasaponin B (**47**) also inhibited the viral VP2 protein expression and inhibition of viral capsid protein synthesis [37].

## 5. Steroids

### 5.1. Steroids Glycosides

Six anti-EV71 saponins, timosaponin B-II (**48**), anemarsaponin II (**49**), timosaponin G (**50**), timosaponin A-III (**51**), timosaponin A-IV (**52**) and shatavarin IV (**53**), were found from the ethanol extract and water extract of *Anemarrhena asphodeloides*. Among these saponins, timosaponin B-II (**48**) displayed a comparable IC_50_ (4.3 ± 2.1 µM) and a 40-fold higher selective index (SI = 92.9) than the positive control (IC_50_ = 361.7 ± 104.6 µM, SI = 2.4) ribavirin [38].

### 5.2. Steroids 

**Figure 6:**
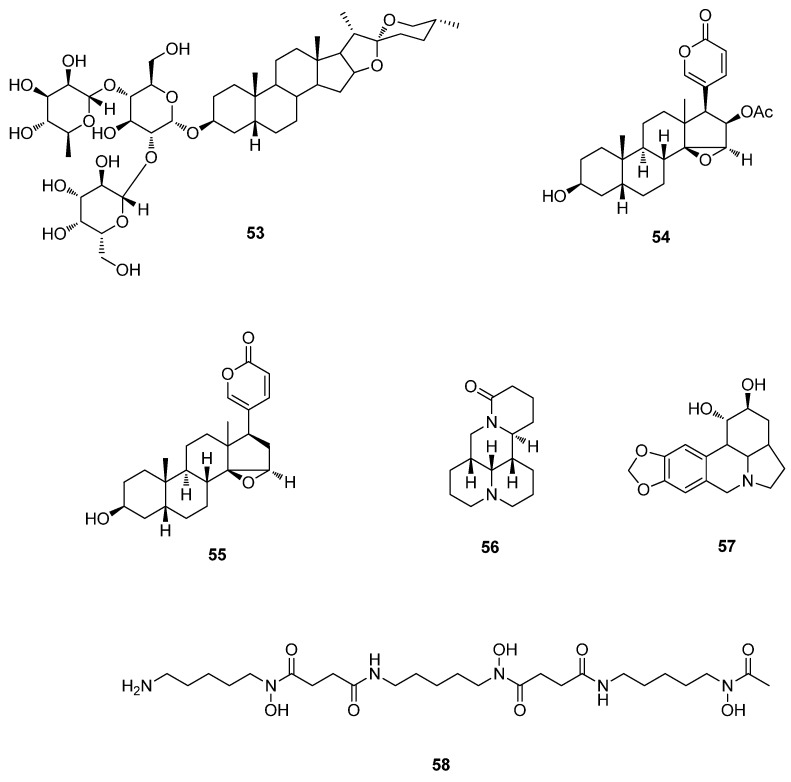
Steroids and alkaloids of **53**–**58**.

Cinobufagin (**54**) and resibufogenin (**55**) were found to inhibit EV71 infection *in vitro* in cell viability and plaque reduction assays. The 50% inhibitory concentrations (IC_50_) of cinobufagin (**54**) and resibufogenin (**55**) were (10.94 ± 2.36) and (218 ± 31) nM, respectively, while their 50% cytotoxic concentrations (CC_50_) were (1277 ± 223) and (1385 ± 254) nM, respectively, and the anti-EV71 selectivity index (SI_50_) of cinobufagin was 116.7, which suggests its potential as a drug. Cinobufagin (**54**) and resibufogenin (**55**) disrupted the synthesis of EV71 protein. However, neither of them inhibited EV71 RNA replication (Figure 6) [39].

## 6. Alkaloids

Matrine (**56**) could suppress the viral RNA on rhabdomyosarcoma cells, reducing the mortality and relieving clinical symptoms [40]. Lycorine (**57**) blocks elongation of the viral polyprotein during translation. Lycorine (**57**) treatment of mice challenged with a lethal dose of EV71 resulted in reductions in mortality, improved clinical scores and fewer pathological changes in the muscles, associated with inhibition of viral replication. When mice were infected with a moderate dose of EV71, lycorine (**57**) treatment prevented paralysis [41]. Deferoxamine (**58**), a marine microbial natural product, compensated for the decreased levels of B cells caused by EV 71 infection. The neutralizing antibody titer was also improved after deferoxamine treatment. Deferoxamine relieved symptoms and reduced mortality and muscle damage, and has the potential for development as a B cell-immunomodulator [42].

## 7. Conclusions

Since the 1980s, EV71 epidemics have occurred in Asian countries and regions, causing a wide range of human diseases. There is no clinical approved antiviral drug currently available for the prevention and treatment of the EV71 viral infections. The few examples of anti-EV71 natural products can be grouped into five main structural classes: flavonoids, polyphenols, terpenoids, steroids and alkaloids. Of all the anti-EV71 NPs, most of them (53/58) have been derived from territorial plants while the remaining five were of marine origins sources. The small number of anti-EV71 NP being discovered over the last 30 years is possibly due to the significant reduction of natural product screening campaigns undertaken by academia and the pharmaceutical industry. One of the technical hurdles is to develop an assay amendable for HTS screening. One plausible reason for the loss in interest by companies is that they cannot patent the structures of NPs. The uniqueness of many NP core structures (or templates) makes these compounds of interest for use as starting points for semi-synthesis and total synthesis. More effort should be put into the screening of NP libraries for anti-EV71 activity.

## Abbreviations

Enterovirus 71	EV71
Natural product	NP
Hand, foot and mouth disease	HFMD
Cytopathic effect	CPE
